# Endovascular repair of infected carotid pseudoaneurysm: A case report

**DOI:** 10.1016/j.ijscr.2020.05.005

**Published:** 2020-05-19

**Authors:** Mohsen Bannazadeh, Ali Reza Sattari, Edvard Skripochnik, Gergios Tzavellas, Apostolos Tassiopoulos

**Affiliations:** Division of Vascular Surgery, Department of Surgery, Stony Brook University, Stony Brook, NY, United States

**Keywords:** Endovascular, Pseudoaneurysm, Carotid endarterectomy, Infection, Case report

## Abstract

**Background:**

Pseudoaneurysm (PA) of the carotid artery is a rare but life-threatening complication following carotid endarterectomy (CEA). Management of carotid PAs is challenging due to the increased risk of stroke and nerve injury in an infected and re-operative field. Open surgery has been the mainstay for this complicated pathology however some patients have characteristics which make an endovascular approach more advantageous. Yet endovascular intervention for infected fields is scrutinized and used as a last option.

**History and Treatment Plan:**

72 year old female with history of basilar artery aneurysm embolization and right internal carotid artery occlusion presented with a left carotid pseudoaneurysm after a CEA 6 months prior. She presented with 2 days of increasing left neck swelling, erythema, and a small ulcerated area with bloody discharge from incision site. A Computed Tomagraphy scan (CTA) showed hematoma surrounding the left ICA concerning for PA. Wound cultures were obtained which grew coagulase (-) staphylococcus. We elected to perform an endovascular procedure to temporize the bleeding by placing a stent graft (7 mm × 7.5 cm Gore Viabahn) across the left ICA. She remains asymptomatic with no recurrent symptoms 6 months postoperatively.

**Conclusion:**

Our experience in this patient indicates that endovascular stenting could be feasible and potentially effective intervention for infection-associated post-CEA PA in patients with an excessively high risk for stroke and nerve injury. We suggest each patient should be evaluated individually and all pertinent characteristics should be considered to make the best decision.

## 1 Introduction

Pseudoaneurysm (PA) after carotid endarterectomy (CEA) is rare but potentially catastrophic. Its incidence has been estimated to be less than 1% [1,2]. Given the significant risk for possible rupture, thrombosis, embolization, and cranial nerve compression, treatment is necessary once PA develops [1,2]. About one third of all post-CEA pseudoaneurysms are associated with local infection. In such situations, the conventional treatment of infection-associated post-CEA pseudoaneurysm has been open surgery which involves PA excision, debridement and arterial reconstruction [1,3]. Recently, endovascular techniques have been utilized to treat post-traumatic and postoperative carotid PA. Endovascular approach is less invasive with a lower risk of immediate stroke or nerve injury, however its durability and feasibility is unknown. We present a case of an infection-associated post-CEA pseudoaneurysm that was repaired successfully at our tertiary academic center using a Gore Viabahn (Flagstaff, AZ, W.L. Gore) covered stent graft. This paper has been reported in line with the SCARE criteria [16].

## 2 Case report

A 72-year-old female presented to our institution with increasing swelling and erythema of the left neck for 2 days associated with purulent discharge from the previous CEA incision. Her past medical history was significant for basilar artery aneurysm embolization 5 years ago. She underwent left CEA with patch angioplasty 6 months prior to presentation. Her right internal carotid artery (ICA) was found to be occluded on prior imaging.

**Fig. 1:**
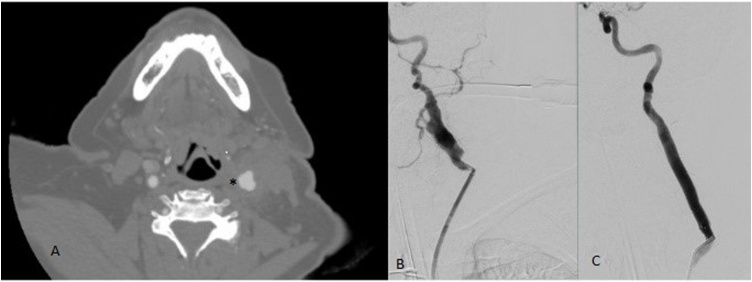
A. Computed tomogram angiography (CTA) showing evidence of left carotid pseudoaneurysm. B. Selective left carotid angiography showing the pseudoaneurysm extending to skall base. C. Successful deployment of left carotid sent graft with no evidence of extravasation.

On physical examination, he was afebrile and hemodynamically acceptable with no leukocytosis. Head and neck exam was significant for pulsatile mildly erythematous mass at the anterior border of sternoclavicular muscle on the left. There was small opening with scant drainage over previous endarterectomy scar. Computed Tomography Angiogram (CTA) of the head and neck was obtained which showed a fluid collection surrounding the left ICA concerning for PA (Fig. 1, asterisk). Wound cultures grew coagulase (-) staphylococcus and the patient was started on intravenous antibiotics for concern of patch blowout. We decided to temporize the PA with a covered stent graft in conjunction with antibiotics. We avoided an open repair due to the risk of nerve injury in a redo neck incision as well as a high risk of stroke due to an occluded right ICA, inaccessible left ICA above the patch and short neck. We suspected that at minimum we would need to sublux the mandible to improve our distal exposure.

Right femoral access was obtained and 90 cm 7 Fr Ansel sheath (Cook. Location) was placed. The left common carotid was selectively catheterized and an 0.018 wire was used to traverse the ICA. A 7 mm × 7.5 cm Gore Viabahn (Flagstaff, AZ, W.L. Gore) stent graft was deployed from common carotid into the proximal ICA with 2 cm coverage of the prior patch both directions. The stent graft was post-dilated with a 7 mm balloon. Completion angiogram revealed brisk flow through the stent graft with no evidence of endoleak (Fig. 1C). The patient was started on dual antiplatelet therapy with Aspirin and Clopidogrel. Repeat Blood cultures were negative and the patient was switched to oral antibiotics for 4 weeks. She was discharged home on postoperative day 10. On 6 month follow up she remains asymptomatic. The patient will be asked to share her perspective on the treatments she received in future visits.

## 3 Discussion

Pseudoaneurysm formation is a rare complication after CEA and is reported to occur less than 1% of the time [1,2,4]. The underlying factors that may cause post-CEA PA development include preoperative infection, hematoma, suture line failure, or patch material degeneration [1,2,4,5]. The consequences of post-CEA PA include possible rupture, ICA thrombosis, cerebral embolization, and cranial nerve compression. About one third of all post-CEA pseudoaneurysms are complicated by infection [1,6,7]. Bovine pericardial patches for CEA have a low incidence of infection which is estimated at about 0.6% [8]. Infection stems from direct extension from adjacent tissues, initial patch contamination, or bacteremia [1,6,7]. Infection-associated post-CEA PA may present days to years following the procedure [1,9] as a localized pulsatile mass with or without pain, draining sinus, abscess, and hemorrhage. Fever, sepsis and stroke are also reported [1,9]. The risk of stroke is about 1%–5.5% following redo CEA [10].

The gold standard treatment of an infected pseudoaneurysm is open surgical repair which involves aneurysmectomy and debridement of infected tissues with vein graft interposition, carotid artery ligation, or extra-anatomic bypass grafting [1,3]. Operating in a field with distorted anatomy due to previous surgery, inaccessible distal endpoint, short neck and occluded contralateral ICA were challenging factors for open surgery in this case. Endovascular treatment for infected carotid aneurysms has been described in the literature [1,9,11] for exceedingly high risk patients. The major benefit of such an approach is to operate under local anesthesia and avoid a re-operative field. This virtually eliminates the risk of cranial nerve injury, which has at least 6% incidence following open surgery [1,12].

Although a controversial topic, the use of covered stents for infected pseudoaneurysms after CEA has gained attraction [1,[12], [13], [14]]. The risk of infecting the graft material is high and theoretically does not allow for definitive treatment. The durability and long-term function of endovascular stenting in these patients is yet to be determined. There is no guideline regarding follow-up but presumably the cornerstone is long-term suppressive antibiotic therapy and surveillance ultrasound or CTA [1,15]. The use of inflammatory markers like ESR, CRP, or blood cultures for surveillance are likely not appropriate [1,15].

Although, there were no clinical signs or symptoms of active infection or stent malfunction in this patient at 6-months follow-up we have committed our patient to lifelong surveillance as there is a risk of recurrent infection. treatment options in the case of recurring infection would be indefinite antibiotic therapy or open repair which probably runs even greater hazard than if performed initially.

Overall, traditional open repair for post-CEA pseudoaneurysm remains the gold standard but endovascular options have gained traction in the literature, albeit guarded. There is no consensus on criteria to choose endovascular over open repair. There are no guidelines on the long-term care or surveillance after endovascular treatment. However, as in prior reports, our experience suggests that an endovascular approach could be feasible and potentially effective intervention for infection-associated post-CEA pseudoaneurysm in a high risk patient.

## Declaration of Competing Interest

None.

## Funding

None.

## Ethical approval

The study is exempt from ethnical approval in our institution.

## Consent

Consent has been obtained.

## Author contribution

Ali Reza Sattari has substantial role in Conceptualization; Data curation; Formal analysis; Investigation; Methodology; Validation; Writing - original draft; Writing - review & editing.

Georgios Tzavellas, has substantial role in Conceptualization; Data curation; Formal analysis; Investigation; Methodology; Validation; Writing - original draft; Writing - review & editing.

Edvard Skripochnik, has substantial role in Conceptualization; Data curation; Formal analysis; Investigation; Methodology; Writing - original draft; Writing - review & editing.

Apostolos K. Tassiopoulos has substantial role in Conceptualization; Data curation; Formal analysis; Investigation; Methodology; Supervision; Validation; Visualization; Writing - original draft; Writing - review & editing.

Mohsen Bannazadeh has substantial role in Conceptualization; Data curation; Formal analysis; Investigation; Methodology; Supervision; Validation; Visualization; Writing - original draft; Writing - review & editing

## Registration of research studies

1. Name of the registry: N/A.
2. Unique identifying number or registration ID: N/A.
3. Hyperlink to your specific registration (must be publicly accessible and will be checked): N/A.

## Guarantor

Mohsen Bannazadeh, MD.

Division of Vascular and Endovascular Surgery Department of Surgery, Health Sciences Center T19-090.

Renaissance School of Medicine at Stony Brook University Stony Brook, NY 11794-8191.

Email: Mohsen.Bannazadeh@stonybrookmedicine.edu.

## Provenance and peer review

Not commissioned, externally peer reviewed.
